# Open Bibliography for Science, Technology, and Medicine

**DOI:** 10.1186/1758-2946-3-47

**Published:** 2011-10-14

**Authors:** Richard Jones, Mark MacGillivray, Peter Murray-Rust, Jim Pitman, Peter Sefton, Ben O'Steen, William Waites

**Affiliations:** 1 Cottage Labs, 13 Brougham Place, Edinburgh, Scotland, EH3 9JX, UK; 2 Open Knowledge Foundation, Cambridge, UK; 3 University of Edinburgh School of Informatics, Edinburgh, UK; 4 Unilever Centre for Molecular Science Informatics, Department of Chemistry, University of Cambridge, Cambridge, UK; 5 Departments of Statistics and Mathematics, University of California, Berkeley, CA, USA; 6 ptsefton, Toowoomba, Queensland, 4350, Australia

## Abstract

The concept of Open Bibliography in science, technology and medicine (STM) is introduced as a combination of Open Source tools, Open specifications and Open bibliographic data. An Openly searchable and navigable network of bibliographic information and associated knowledge representations, a Bibliographic Knowledge Network, across all branches of Science, Technology and Medicine, has been designed and initiated. For this large scale endeavour, the engagement and cooperation of the multiple stakeholders in STM publishing - authors, librarians, publishers and administrators - is sought.

## 

BibJSON, a simple structured text data format (informed by BibTex, Dublin Core, PRISM and JSON) suitable for both serialisation and storage of large quantities of bibliographic data is presented. BibJSON, and companion bibliographic software systems BibServer and OpenBiblio promote the quantity and quality of Openly available bibliographic data, and encourage the development of improved algorithms and services for processing the wealth of information and knowledge embedded in bibliographic data across all fields of scholarship.

Major providers of bibliographic information have joined in promoting the concept of Open Bibliography and in working together to create prototype nodes for the Bibliographic Knowledge Network. These contributions include large-scale content from PubMed and ArXiv, data available from Open Access publishers, and bibliographic collections generated by the members of the project. The concept of a distributed bibliography (BibSoup) is explored.

## Technical note

This paper was created using the technologies described in the text. All bibliographic entry references and bibliographic entries were managed in BibJSON then included in the HTML document following the Scholarly HTML convention. The document itself is formally consistent with these specifications and can be read as a normal HTML document. It would alternatively be possible to embed bibliographic records in the document directly from BibJSON via JavaScript The "flat HTML" should be taken as the definitive version, and can be re-purposed into other formats (Additional file 1).

## Competing interests

The authors declare that they have no competing interests.

## Authors' contributions

All authors took equal parts in creating the concepts and tools reported and all authors wrote and revised the manuscript.

## Supplementary Material

Additional file 1 **HTML document**. The complete HTML document of this manuscript. Click here for file

