# Supplementary material for: Open Bibliography for Science, Technology, and Medicine
Source: J Cheminform. 2011 Oct 14;3:47. doi: 10.1186/1758-2946-3-47 (PMC3206455; doi:10.1186/1758-2946-3-47)
Supplement: Additional file 1 — HTML document. The complete HTML document of this manuscript. [file 1758-2946-3-47-S1.HTML]

# Open Bibliography for Science, Technology, and Medicine

Authors/Affiliations:

- Richard Jones / Cottage Labs
- Mark MacGillivray / Open Knowledge Foundation / University of Edinburgh School of Informatics / Cottage Labs
- Peter Murray-Rust / Unilever Centre for Molecular Science Informatics, Department of Chemistry, University of Cambridge
- Jim Pitman / Departments of Statistics and Mathematics, University of California, Berkeley
- Peter Sefton / ptsefton.com
- Ben O'Steen / Cottage Labs
- William Waites / Open Knowledge Foundation / University of Edinburgh School of Informatics

## ABSTRACT

The concept of Open Bibliography in science, technology and medicine (STM) is introduced as a combination of Open Source tools, Open specifications and Open bibliographic data. An Openly searchable and navigable network of bibliographic information and associated knowledge representations, a Bibliographic Knowledge Network, across all branches of Science, Technology and Medicine, has been designed and initiated. For this large scale endeavour, the engagement and cooperation of the multiple stakeholders in STM publishing – authors, librarians, publishers and administrators – is sought.

BibJSON, a simple structured text data format (informed by BibTex,
Dublin Core, PRISM and JSON) suitable for both serialisation and storage of large quantities of bibliographic data
is presented. BibJSON, and companion bibliographic software systems BibServer and OpenBiblio promote the quantity
and quality of Openly available bibliographic data, and encourage the development of improved algorithms and
services for processing the wealth of information and knowledge embedded in bibliographic data across all
fields of scholarship.

Major providers of bibliographic information have joined
in promoting the concept of Open Bibliography and in working together to create prototype nodes for the
Bibliographic Knowledge Network. These contributions include large-scale content from PubMed and ArXiv, data
available from Open Access publishers, and bibliographic collections generated by the members of the project. The
concept of a distributed bibliography (BibSoup) is explored.

## 0 TECHNICAL NOTE

This paper was created using the technologies described in the text. All bibliographic entry
references and bibliographic entries were managed in BibJSON then included in the HTML document
following the Scholarly HTML convention. The document itself is formally consistent with these specifications and
can be read as a normal HTML document. It would alternatively be possible to embed bibliographic records in the
document directly from BibJSON via JavaScript The "flat HTML" should be taken as the definitive version, and can be
re-purposed into other formats.

## 1 INTRODUCTION

We introduce the concept of Open Bibliography as a combination of
Open Source tools, Open specifications and Open bibliographic data. Our
Open Bibliography project is an umbrella of several other initiatives,
most prominently the Open Knowledge Foundation’s Bibliographic Working
Group [1], the JISC-funded JISC-OpenBib project at the University of Cambridge
[2], and the NSF-funded Bibliographic Knowledge Network project
[3].
These projects have all addressed the totality of Open bibliographic
resources including design of systems, implementation of software,
licenses for use and re-use, and the collection and hosting of
substantial bibliographic datasets. In this article we shall concentrate
on bibliographic data for articles in the Science, Technology and Medicine
(STM) fields, but we introduce the reader to the wider elements of
bibliography before the main results. We stress that the tools and
formats exemplified here have a particularly simple modular form in STM
article publishing; however, these tools and formats are designed to be
both flexible and extensible, and are also capable of managing library
and personal collections, monographs, multiple versions *etc.*

Bibliographic records are traditionally held as 3 X 5 cards in a library card catalogue, and more recently represented in languages and syntaxes such as MARC, BibTeX, RIS [4] [5] [6]
[7], and a variety of meta-languages such as XML and JSON [8] [9]. This data has been commoditised, and is subject to a large scale cycle of use (publication / collection / abstracting / indexing / searching / citation) which involves all participants in scientific publication: authors, librarians, publishers and administrators.

Bibliographic data can be presented on various scales (such as individual
records, publication lists, departmental collections, subject-specific
repositories, and the databases behind large scale indexing services). But, bibliographic data is
subject to a process of continual creation and replication; the elements of bibliographic data are facts, which in most jurisdictions cannot be
copyrighted. In some jurisdictions it is possible to copyright the expression of facts and the European sui generic directive allows protection of collections of facts. The intention of Open bibliography is to provide a substantial and growing bibliographic collcection in the public domain which will remove the need to negotiate any Intellectual property relating to bibliography

There are few technical and legal obstacles to widespread
replication of bibliographic records on a massive scale – the main
limitations of such activity are social: whether individuals and
organisations are adequately motivated to create and maintain open
bibliographic resources. But the dynamics of creation and replication of
bibliographic records have been irreversibly changed by recent
technological and social developments, most notably the emergence of:

- UTF-8 as a universal format capable of encoding characters in all languages
- XML and JSON as ubiquitous standards for encapsulating and serializing text data records for
  transmission over the internet
- the internet-based architecture of web services such as RESTful APIs [10]
- NoSQL [11] database software such as CouchDB [12],
  and Open Source indexing systems based on Lucene [13] such as SOLR
  [14] and ElasticSearch [15], which greatly
  facilitate bibliographic data storage and retrieval
- the global Open movement where governments, funders and individuals
  recognise the value of Open information, and have begun developing the
  technical and cultural requirements to support it [16] [17] [18] [19]

Bibliographic data has long been understood to contain important
information about the the influence and impact of various authors and
journals on scientific disciplines [20] [21]. However, now, instead of privileged data owners building proprietary reputation factors on the basis of
large proprietary but incomplete bibliographic data stores [22], Open Bibliography would allow those
concerned to run their own reputation algorithms across an ever-larger Open
data store; they could also submit complex queries, make selections and
analyses of the Bib data to create collaboration graphs, etc.

The tools for such easy processing are not yet adequately developed,
so our aim is to provide Open tools and services to make the wealth of
bibliographic data available to the widest possible audience, and to
promote increased understanding of science and technology, especially in
interdisciplinary areas.

## 2 SCOPE

### Bibliography

Traditionally, “bibliography” has been regarded as the study of library
holdings and catalogues, and, more recently, catalogues of material
published by formal publishers, repositories and other collections. We
wish therefore to explain the importance of bibliography to scientists,
and to argue the merits of Open Bibliography, by which we mean
systematic efforts to create and maintain stores of Openly accessible [23],
machine-readable bibliographic data.

The unit of bibliography is the bibliographic record, which consists
of the information necessary to locate and/or identify a
publication (and, increasingly, other resources besides textual
material, such as authors, images and scientific datasets). The term
“bibliography” is also often used to represent a personal collection of
bibliographic records (and in some cases is synonymous with citation
lists). We refer to such a collection here as a “bibliographic dataset”.

We use the term “citation” to mean a reference to a bibliographic
record within the body of a document. A citation may also be called
simply a “reference”. We will not say much more about citations, except
to point out that an improved approach to bibliography should also be of
value to citation management and analysis. Open Bibliography can exists today for all scholarship. Open citations can only exist for a small subset.

Despite the importance of bibliography, including the widespread sale
of bibliographic records, there is no single syntax or agreed semantics
for the publication and exchange of STM bibliographic records;
scientists use whatever representation is provided by their tools, for example BibTeX from the LaTeX [24]
authoring system; the publishing community commonly uses PRISM,
although some publishers have their own representation of their
bibliographic data, which often consists of a mixture of Dublin Core [25],
PRISM, BibTeX and their own markup approach.

### Community

We are using “Open Bibliography” to represent an open philosophy for managing bibliographic objects. In some cases, open bibliography will refer to collections of bibliographic objects, but we do not imply that there must be a single central authoritative resource. In order to support sharing of bibliographic records across sites, we are developing BibJSON with a view to creating a BibSoup -a distributed collection of bibliographic records in a simple, lightweight and portable format. We expect that the first stage, at least, will be the identification of bibliographic collections which are Open and where the collectors can offer them with the appropriate common technology. The community of groups committing to providing open bibliographic collections, with some cooperation about formats and interfaces, is what we mean by the Bibliographic Knowledge Network.

We are aware of other groups offering large amounts of bibliographic data, operating under open licences, such as K4All [26] and Wikipedia [27]. There are also a substantial
number of large bibliographic repositories which are operationally
fairly Open, even if the data is not explicitly declared to be so, or
available only through an API rather than in bulk, such as arXiv, RePEc,
BibSonomy, PhilPapers, DBLP, CiteULike, Connotea, Zotero, Mendeley *etc.* [28]
[29] [30]
[31] [32]
[33] [34]
[35] [36].
We have worked closely with two major Open Access publishers (International Union of Crystallography (IUCr) and
BioMed Central (BMC)) [37]
[38], and have collaborated with PubMed [39],
with Thomas Krichel’s AuthorClaim [40] and 3Lib [41] projects, and with the Sciplore team [42].

### BibSoup recipe for Science, Technology and Medicine

Because this article is limited to exploring bibliography for STM, we
have taken a pragmatically simple approach. Approaches such as FRBR [43] and
BIBO [44]
make provision for complex aspects of bibliography such as multiple
manifestations and representations of works and multiple versions. While
these are relevant to STM bibliography in some areas [45], for the most part we
do not need the complexity of these and other RDF [46] approaches, although our
tools and software should be capable of leveraging them if necessary.

We report a number of prototypes in both tools and collections, and
also propose that STM bibliography can be adequately represented for
most immediate purposes using BibJSON [47].
Due to the intense interest in Open Bibliography, we are now very
actively working on future versions of BibJSON, but the examples given
in this article are fully supported by current software. In the spirit
of the “perpetual beta” approach on the web, we intend to release early
and often in public view so that a broad community becomes intimately
involved in the design of specifications. As a first example, the
references in this document are stored in a prototype of BibJSON and can be
rendered into the content via JavaScript.

We accept that our approach may not be suitable for the industrious world of library management, and may be seen as something of the level of cooking round the campfire. However, our main aim in this instance is to provide a way for people to easily represent and share their bibliographic records and collections, firstly in the Science, Technology and Medicine domain, by achieving the following:

1. Specify a simple common syntax – BibJSON
2. Build a few useful collections
3. Help others build up collections by converting from other formats
4. Share those collections via APIs
5. Thus creating the BibSoup

## 3 MOTIVATIONS FOR OPEN BIBLIOGRAPHY

We present in this section a list of reasons and use cases which motivate our commitment to Open Bibliography.

1. **Access to Information.** There is currently no single
   place where a user can obtain a definitive statement of the identity
   and public domain components of a bibliographic record in STM
   publications. There are a number of organisations, many commercial,
   which supply bibliographic records but almost all of these are covered
   by licences which limit their re-use. This means, for example, that
   users cannot easily compare records from different suppliers, nor can
   these records be integrated into a single definitive resource. By
   contrast the idea of Open Bibliography is to empower and encourage
   individuals and organisations of various sizes to contribute, edit,
   improve, link to and enhance the value of public domain bibliographic
   records.
2. **Error detection and correction.** We expect that, as for resources like Wikipedia and Open
   StreetMap [48],
   the community supporting the practice of Open Bibliography will rapidly
   add adequate means of checking and validating the quality of openly
   accessible bibliographic data. Errors in bibliographic data are common,
   and an Open approach allows for crowd-sourcing detection and correction
   of errors. In some cases this may be done by individuals (*e.g.* in Open
   StreetMap or ChemSpider [49]) and in other cases may be through organisations
   which appreciate Open Bibliography and contribute updates to it.
3. **Publication of small bibliographic datasets.** It is
   common for individuals, departments and organisations to provide
   definitive lists of bibliographic records. Examples of these are reading
   lists produced by a lecturer, study lists created by students in the
   course of their studies, publications lists created by researchers and
   departmental and institutional lists reflecting on the work published
   from those organisations. The practice of Open Bibliography encourages
   individuals and small organisations to make such lists available as a
   shared, machine-readable resource. These lists then contribute to the
   quality of the open bibliographic aggregation, and reduce the effort of
   aggregating agents in compiling lists across a number of individuals or
   departments. RePEc provides the leading example of feasibility of this
   sort of bibliographic aggregation for a subject community.
4. **Merging bibliographic collections.** We show here
   that Open Bibliographic collections will come from different subject
   groups *e.g.* bioscience, crystallography and mathematics. Sometimes there
   will be large overlap and sometimes the resources will be largely
   independent. In the next period of work we intend to create a merged
   bibliographic resource, BibSoup [50],
   as an aggregation of Open collections which can be readily queried to
   return basic bibliographic information in machine-readable format
   suitable for further processing. We do not expect an Open BibSoup to
   replace massive central search systems such as Google Scholar and
   Microsoft Academic Search which require considerable infrastructure to
   host and maintain. Rather, we expect that once a result set has been
   obtained from these or other search services, it will be possible to link the BibSoup result set to useful services. These services should return further information about results, especially community-validated machine-readable metadata for further
   use and processing, something currently unavailable from any large scale
   search service. Thus BibSoup implementations could take advantage of
   the work already done by Google [51], Microsoft and other search providers,
   to increase discoverability by improving the quality and ranking of search results.
5. **A bibliographic node in the Linked Open Data cloud.**
   There are many reasons why the world may wish to discover STM
   bibliography and to link to it. For example, many Wikipedia articles
   cite STM publications and it would be valuable to know whether these
   exist, to obtain complete bibliographic metadata for referencing, and to
   know whether they can be read and re-used without permission.
   Communities can add their own linked and annotated bibliographic
   material to the LOD cloud [52].
6. **Collaboration with other bibliographic organisations.**
   Many resources in academia are collected by and supplied by commercial
   organisations on a service basis. We expect this to continue and we
   offer the products of Open Bibliography as resources against which these
   suppliers can validate and compare their offerings. Examples of such
   organisations are reference manager suppliers (Zotero, Mendeley, EndNote [53]),
   reference and identifier systems such as CrossRef [54]
   [55], and academic libraries and library organisations.
7. **Mapping scholarly research and activity.**
   Bibliographic records (including citations) are now frequently used for assessing the value of individuals and institutions. Open
   Bibliography can provide definitive records against which these
   assessments can be collated. For example it allows us to create patterns
   of collaboration and to identify geographical locations in which work
   is performed. For researchers in this area, we expect the type of
   analysis shown in our geospatial examples to be of broad interest (even
   though the citations and abstracts may not always be Open).
8. **An Open catalogue of Open scholarship.** Since the
   bibliographic record for an article is Open, it can be annotated to show
   the Openness of the article itself, thus bibliographic data can be
   Openly enhanced to show whether a paper is fully Open (*e.g.* CC-BY),
   freely-available (as in beer), and the website it was discovered, and
   the association of non-textual objects such as datasets, multimedia and
   other resources. Open bibliographic data can also include syntactic
   metadata such as the format size and technical accessibility of the
   resources. Beyond this, we believe that a large number of hitherto
   unpublished applications can be made on top of an Open bibliographic
   framework.
9. **Cataloguing diverse materials related to bibliographic records.**
   We see the opportunity to list databases, websites, review articles and
   other information which the community may find valuable, and to
   associate such lists with open bibliographic records.
10. **Use and development of machine learning methods for bibliographic data processing.**
    Widespread availability of Open Bibliographic data in machine-readable
    formats should rapidly promote the use and development of machine
    learning algorithms, allowing machines to largely automate tasks such as
    matching, de-duplication and classification of bibliographic records,
    and to make Open Source versions of these algorithms widely available
    for use by managers of Open Bibliographic data stores.
11. **Promotion of community information services.**
    Widespread availability of Open Bibliographic web services will make it
    easier for those interested in promoting the development of scientific
    communities to develop and maintain subject specific community
    information services, featuring searchable lists of books, articles and
    web resources of interest to a community of practice. Every such service
    may be thought of as a node in the Bibliographic Knowledge Network, a
    node which acquires, refines and organises data from the larger BibSoup
    environment, and publishes this data Openly back to the network.
12. **Reputation management and the citation graph**
    We expect a major outcome of Open Bibliography will be the ability to build better citation lists and graphs. Unfortunately most citations are not yet Open. We urge those who are creating Open citation lists whether as part of the publication or as a collection to cast the syntax and semantics in BibJSON.

## 4 DEFINING BIBLIOGRAPHIC RECORDS

Bibliography, and bibliographic data, is sometimes regarded as
referring to everything that is not part of the “full text” and “images”
in an article. This can be problematic because some people and
organisations regard material such as abstracts, annotations and
citation lists as “copyrightable” and therefore not by default Open. In
this article, we do not debate the ethics and legality of asserting
ownership over certain types of bibliographic data, and our
understanding of the agreed law and practice is that what we define as
“core bibliographic data” below can be made Open by default.

By “core bibliographic data” we mean that data which is necessary to
identify and / or discover a publication. It is generally held that such
bibliographic data is NOT copyrightable and this has been confirmed by
the Association of STM Publishers in a public reply to one of the
authors [56].

It is difficult to get authoritative statements as to whether other
fields are Open by default. But we would expect, for example, that the
format of the work and the rights associated with it were by default
Open, while the abstract and images were not. Traditionally collections
of STM bibliographic data have been expensive to produce and most of
these are therefore currently available only under licenses that
restrict re-use. Because it is now technically possible to create large
amounts of Open Bibliographic data, this opens the possibility of
collections created from the start as Open and distributed for community
re-use.

The following “core bibliographic data”, as described by the Open Bibliography Principles
[57], will be the subject of this article:

- names and identifiers of author(s) and editor(s)
- titles
- publisher information
- publication date and place
- identification of parent work (*e.g.* a journal)
- page information
- URIs

A number of ways of creating Open Bibliographic data may be identified:

1. Contributions from a publishing agency under an Open license such as
   CC0 or PDDL (effectively putting the material into the public domain)
   or CC-BY which allows use of the data in exchange for links back to the
   source (especially suitable for data elements such as abstracts)
2. Collections from Open Access digital repositories
3. Collections developed by spidering the web and extracting public
   domain bibliographic data components from publication lists, in the
   manner of CiteSeer [58]
4. Donations of data by individual researchers, departments and universities
5. Donations from publishers of collected scientific information such as Medline [59]

Using these and other mechanisms, we believe that is it compelling to create and maintain an Open bibliographic network of
information about STM publications. These need not necessarily be
electronic publications but the stress of this article will be on the
collection of bibliographic data that refers to electronic journals, web
pages, technical reports, theses, and documents available on the web,
meaning the data that is required to locate and identify a document on
the web, whether or not the full text of that document may is openly
available. As an example, it is possible to extract bibliographic data
for all the publications in the BMC collection of journals. The web has
been crawled for many years and the technology for doing this is
standard. It is polite, but not legally required, to agree large-scale
crawling with a publisher or to create web-server-friendly robots which
do not impose undue stress.

## 5 REPRESENTING BIBLIOGRAPHIC RECORDS

Most scientists require a single bibliographic record per
publication. In other words, most scientists do not distinguish between a
print version, an electronic version or a manuscript on an author’s web
page or in their institutional repository. Scientists have the implicit
model of a single platonic bibliographic record for an article. Our
approach is based on this and while there may be occasional complexities
that cannot be represented, we believe it is powerful enough to create a
useful sustainable Open STM bibliography.

### Vocabulary

The vocabulary terms used by publishers and other bibliography
creators, often drawn from Dublin Core, PRISM, Medline or home grown
element sets, are fairly, but not completely, interchangeable. For
example. dc:creator might be used for authors in one source and editors
or publishers in other sources, but usage is normally consistent within a
given source. As a first step we propose to honour the terms used by
the collectors rather than attempt to align and normalise them
algorithmically. We are exploring whether there is a pragmatic
“flattening” of the main concepts and whether it is possible to manage
“most” STM bibliographic records with a small number of central terms;
most STM articles in journals can be described with a very small subset
of these vocabularies:

| Types of entities | | |
| --- | --- | --- |
| Name | Element Set(s) | Description |
| Agent | FOAF, dcterms | A resource that acts or has the power to act. |
| Person | FOAF | A person |
| Organisation | FOAF | An organisation |
| Document, Bibliographic Resource | FOAF, BIBO, dcterms | A document of some sort |
| Article | BIBO | An article, typically in a Journal |
| Issue | BIBO, BibTeX | A journal issue or volume (expressed as a property in BibTeX, linked with dcterms:isPartOf in BIBO) |
| Journal | BIBO, BibTeX | A journal (expressed as a property in BibTeX, linked with dcterms:isPartOf in BIBO) |

| Properties or predicates | | |
| --- | --- | --- |
| Name | Element Set(s) | Description |
| author, creator, contributor,editor | BibTeX, dcterms | Person or organisation creatively responsible for some document |
| identifier | BibTeX, BIBO, dcterms | Identifier of an entity such as an article or journal (including refinements such as ISSN, DOI, ISBN, *etc.* which are common BibTeX extensions) |
| institution | BibTeX | The institution involved in publishing |
| journal | BibTeX | A journal (see Journal in classes above) |
| month, year, published | BibTeX, dcterms | The date of publication |
| name, label | FOAF, RDFS, SKOS [60] | A name or label for a thing such as a person or organisation. |
| pages, extent | BibTeX, BIBO, dcterms | Page numbers |
| publisher | BibTeX, dcterms | A publisher |
| title | BibTeX, dcterms | The title of the work |
| volume | BibTeX | The volume of a journal (see Issue in classes above) |

We could equally well have included the relevant fields from MARC21 and more in the above table. What these representations, MARC21, BibTeX and
BIBO+dcterms, have in common is a flat representation of a bibliographic
record. This flat representation is a core feature of our conceptual model.

### Identifiers

Identifiers are critically important. They are necessary (but
obviously not sufficient) to enable tasks like de-duplication – in order
to identify duplicates, we need to be able to identify the things that
are duplicated. They also make it possible to refer to entities outside
of the current dataset; one might refer to the author of an article by
their Wikipedia page, for example. This is not necessary, but it opens
up many interesting possibilities for interlinking and correlating
amongst datasets. Using a URI as an identifier where feasible is
therefore a desirable feature [61].

Where a single, sustainable resource manages bibliographic data, it
makes sense for it to generate its own unique identifiers, even if there
is already a well-defined identifier system for some of the
information. Thus, in working with the British Library [62] on the British
National Bibliography [63],
we have created a set of identifiers for their records. However, where
collections come from several sources it is very difficult to create a
global unique identifier system without a curating organisation. We
therefore expect that each collection will create its own identifiers.
We expect that different collections will contain bibliographic data for
the same object and here we will create a mapping between the
collections rather than trying to create a single global index.

### Datasets

We have also worked with the following datasets (see also Section 6)
and found that the records can be well represented by the concepts
above.

1. Bibliography extracted from the masthead (splash page) of 8000 Open
   Access articles from the IUCr. These already contain bibliographic
   information in PRISM and Dublin Core, together with some submitted by
   authors (*e.g.* email and addresses).
2. The Open Access subset of PubMed Central (PMC). There are about 250,000 fulltext
   papers which contain bibliographic data but which vary due to the
   publishers’ syntax and semantics. These have been normalised so that the
   information is in a uniform schema, but publisher variation still
   exists in terms of metadata quality and how key information like DOIs
   and identifiers are represented.
3. Recently we have obtained the full bibliographic records for 20
   million Medline articles with metadata defined in the National Library of Medicine(NLM) Medline DTD.
4. Personal bibliographies of about a hundred researchers in the fields
   of mathematics and statistics, including all Mathematics Faculty at U.
   C. Berkeley [64].
5. Various lists of authors in mathematics, statistics and related fields [65] .

### Serialisation

With this conceptual model in hand, we can turn our attention to
exchanging information between systems that have similar or at least
compatible models. For pragmatic reasons we propose to use JSON to
exchange this information. JSON is widely implemented, simple to parse
and easy to create either with a computer program or by hand in a text
editor. A JSON-based format which uses dictionaries or associative
arrays is also extensible since adding a new key to such a dictionary
should not break any existing implementations which may not understand
the meaning of the new key. JSON also has the advantage over XML of underpinning many web services – it exists specifically to pass data back and fore between javascript code; it fits well with our software development goals.

By using a JSON-based format designed for representation of
bibliographic data – meaning data about documents of various kinds, and
about the people, organizations and subjects connected to those
documents – we can include guidance for creating records, linkages to
existing ontologies, vocabularies and schema, and schema definitions,
covering a wide range of bibliographic needs and drawing from a number
of bibliographic metadata sources (BIBO, BibTeX, DC). If desired, a
creator of a bibliographic dataset may add more information (*e.g.*
language, format, editors, *etc.*). A consumer of this dataset may or may
not read and understand this.

## 6 IMPLEMENTATIONS

This paper is strongly informed by the work done in the JISC-OpenBib project, a collaboration between the
University of Cambridge, the OKF, the British Library, Cambridge University Library and the IUCr. With the help
of these partners, the high-level goal was to take exemplary bibliographic datasets and show that the
principles of Open Bibliography, coupled with the formalisation and tools reported here, would be of great value
to the scientific and informatics communities. We report a number of successful prototypes in this section where
we have been able to acquire or collect an Open dataset, transform it to BibJSON or equivalent and re-purpose it,
often with an interactive tool. The emphasis in this paper is on STM bibliographic resources but for completeness
we also report on other collections.

### Bibliographica

The OKF has developed a bibliographic management system (Bibliographica) which was functional at the start of this
project. Although general, it has been primarily aimed at non-STM resources such as library collections and
personal bibliographies. During the project, the British Library released under a CC0 licence the British National
Bibliography (a collection of about 3 million records for monographs created in their role as deposit library).
These have been converted to as queryable RDF using our Open Source OpenBiblio software
[66] [67].
This provides an example of using the software to make bibliographic metadata available as RDF where required;
this and other instantiations of OpenBiblio then act as resources for building bibliographic collections.

### Medline

The largest collection of STM bibliographic data is provided by the NLM from the
National Institutes of Health (NIH) of the USA. This is provided freely, and the records refer to both Open and
non-Open publications. The Open publications are referred to as the “Open Access subset (OAS)” (the terminology is
complex and our project has explained it [68] ).
The OAS (*ca.* 250,000 records) contains full text
and full reference lists (citations), and is of a very tractable size for carrying out prototypic work on
bibliography and citations. The full Medline collection has about 20 million articles and in collaboration
with the NLM we have obtained these records and converted them to RDF using a straightforward BIBO+dcterms
representation. For the full record set we have been careful to include only those components of bibliography
which are agreed to be Open (*i.e.* we have omitted abstracts and editorial annotation). Nevertheless, this
collection is a major new resource in Open Scholarship.

We have converted both subsets to RDF, and found that while the Open subset is tractable with a wide range of
common tools, the full records have problems of scale. It produced over 1 billion RDF statements; the resources
required for querying this in an RDF store are beyond current scope, however a sample record is appended, and
further information along with full content is Openly available [69] [70].
This problem of RDF scalability is being tackled by commercial creators of triple stores, and we expect the technology to evolve to support this. For the moment, however, we are making progress with the BibJSON approach and storing the records in a NoSQL database (CouchDB). This gives good performance for the sorts of queries that most people will initially wish to make, at the expense of losing SPARQL querying.

Despite not having the abstracts or full text Openly, the Medline bibliographic dataset has still enormous value,
particularly when used with new ways of navigation and display.

Although citations (bibliographic entry reference lists) are outside the scope of Open Bibliography, the OAS
provides an opportunity to work on citations. This is less easy because the reporting of citations is poorly
formalised (a major motivation for Open Bibliography and BibJSON), and they contain a large number of errors,
including non-existent bibliographic objects. However, the potential is large and we display an analysis of
citations related to a retracted paper [71].

Figure 1: A citation map of papers recursively referencing Wakefield’s paper on the adverse effects of MMR vaccination.
Note that this graph has been built manually, systems with typed citations such as CITO [72] would allow it to be created automatically. A full analysis requires not just the act of citation but the sentiment, and initial inspection shows that the
immediate papers had a negative sentiment *i.e.* were critical of the paper. Wakefield’s paper was eventually withdrawn but the other papers in the map still exist. It should be noted that recursive citation can often build a false sense of value for a distantly-cited object.

### Visualisations

Traditionally, bibliographic records have been seen as a management tool for physical and electronic collections,
whether institutional or personal. In bulk, however, they are much richer than that because they can be linked,
without violation of rights, to a variety of other information. The primary objective axes are:

1. Authors. As well as using individual authors as nodes in a bibliographic map, we can create co-occurrence of
   authors (collaborations).
2. Authors’ affiliation. Most bibliographic references will now allow direct or indirect identification of the
   authors’ affiliation, especially the employing institution. We can use heuristics to determine where the bulk
   of the work might have been done (*e.g.* first authorship, commonality of themes in related papers *etc.*
   Disambiguation of institutions is generally easier than for authors, as there is a smaller number and there
   are also high-quality sites on the web (*e.g.* wikipedia for universities). In general therefore, we can geo-locate all the components of a bibliographic record.
3. Time. The time of publication is well-recorded and although this may not always indicate when the work was
   done, the pressure of modern science indicates that in many cases bibliography provides a fairly accurate snapshot
   of current research (*i.e.* with a delay of perhaps one year).
4. Subject. Although we cannot rely on access to abstracts (most are closed), the title is Open and in many
   subjects gives high precision and recall. Currently, our best examples are in infectious diseases, where terms
   such as malaria, plasmodium *etc.* are regularly and consistently used.

With these components, it is possible to create a living map of scholarship, and we show two examples carried out
with our bibliographic sets.

Figure 2: This is a geo-temporal bibliographic map for crystallography. The IUCr’s Open Access articles are an
excellent resource as their bibliography is well-defined and the authors and affiliations well-identified. The
records are plotted here on an interactive map where a slider determines the current timeslice and
plots each week’s publications on a map of the world. Each publication is linked back to the original article. (The
full interactive resource is available at http://benosteen.com/timemap/index.)

Figure 3: This is a geo-temporal bibliography from the full Medline dataset. Bibliographic records have been extracted
by year and geo-spatial co-ordinates located on a grid. The frequency of publications in each grid square is
represented by vertical bars. (Note: Only a proportion of the entries in the full dataset have been used and
readers should not draw serious conclusions from this prototype). (A demonstration screencast is available at http://vimeo.com/benosteen/medline; the full interactive resource is accessible with Firefox 4 or Google Chrome, at http://benosteen.com/globe.)

These visualisations show independent publications, but when the semantic facets on the data have been extracted
it will be straightforward to aggregate by region, by date and to create linkages between locations.

### Mashups

Bibliographic data are particularly valuable for mashups (*i.e.* the combination of data components that
share one or more common values or identifiers). Thus, for example, it should be possible to link Open Bibliography
to bibliographic entry references in Wikipedia. More generally, Open Bibliography is available for any author or
organisation who wishes a definitive identification of the bibliographic entry references in a document.

Our mashups demonstrate that when data is openly available, it enables serendipitous and relatively quick
development of useful tools. For example, we created a Wikipedia bookmarklet, a personal collections tool on
Bibliographica, and a relevant reading list generator for the Edinburgh International Science Festival
[73] [74] [75].

### BibJSON-based collections and systems

In order to manage larger bibliographic datasets in a simpler format, we are now collaborating with Bibliographic
Knowledge Network to develop the BibJSON format for representing bibliographic records. It is sufficient for most
current purposes for basic STM articles, adequate also for all basic BibTeX types including monographs, and is
extensible so it can easily support records for authors, journals, *etc.* The main virtues are:

- it is simple to understand.
- it can support conversion from all the common formats.
- it is easy to install and run software which processes it with added value to the user.
- it unifies the current fragmentation of *ad hoc* exports utilized by STM publishers.
- it is an Open format supported with Open Source tools.

We have created sample BibJSON conversions using our software
(examples appended), and will continue to perform these conversions on
the datasets now available. BibJSON can perform a similar function for
communities wishing to share bibliographic data as GeoJSON [76] does for
those sharing geospatial information.

### BibSoup

We take the view that any Open bibliographic record (with its provenance) is
potentially valuable, even though there may be duplicates referring to “the same bibliographic object”. By representing these records in the simple format of BibJSON allows us to build from the bottom up a collection of all references that people are interested in sharing – the BibSoup. The question
of determining whether two records relate to “the same object” is difficult and controversial and BibSoup
deliberately avoids this. It consists of a number of collections of bibliography (initially article metadata in STM areas) united
by a common syntax. It is left to humans and machines to develop annotations and equalities between the components
of these collections. Thus, for example, “the same paper” will be reported in arXiv, DBLP and possibly even
Medline.

The BibSoup approach encourages the contribution of Open bibliography without complexity at
contribution time, helping to overcome the hurdle of community engagement. We take the mess of current STM bibliography as a starting point and, where the community commitment, political will and financial support is available, offers methods for tidying this up.

## 7 FUTURE WORK

Via collaboration with the Scholarly HTML (ScHTML) [77]
community we intend to follow conventions for embedding bibliographic
metadata within HTML documents whilst also enabling collection of such
embedded records into BibJSON and BibSoup, thus allowing embedded
metadata whilst also providing additional functionality such as search.
We are also working towards ensuring compatibility between ScHTML and
Schema.org [78], affording greater relevance and usability of ScHTML data.

We are continuing development of BibServer [79]
along with the BibJSON specification as a way for individuals – or
departments or research groups – to easily manage, present, and search
their own bibliographic collections. Collections can be stored in BibTex
files, in JSON files or a JSON database such as CouchDB, or in an
OpenBiblio instance, or managed directly by the software. The key to the
architectural design is that it will be possible for other interested
parties to develop their own plugins both for ingest and storage,
allowing flexibility in implementation; complexity is somewhat reduced by focussing on published serial articles for now.

We are in the early days of proving concept; however the biggest challenge lies ahead – and that is sustainability. This is not something we can achieve on our own, and we are actively seeking collaboration with major stakeholders – libraries, funders, researchers – whilst submitting funding proposals aimed at developing sustainability.

These ongoing efforts to develop OpenBiblio, BibJSON and BibServer,
will enable us to support large scale Open Bibliographic data – the
BibSoup. We hope to attract further collaborations from other groups
which realise the importance of Open Source code, Open Data and Open
Knowledge to the future of scholarship.

## 8 APPENDIX

### 8.1 Bibliographic records represented in BibJSON

The following examples demonstrate conversions of typical
bibliographic records into BibJSON. Although BibJSON is not a complete
standard, the aim is to demonstrate the simplicity with which we can
represent this data in a JSON object, using namespaces to extend keys as
necessary. The default namespace for BibJSON keys is essentially BibTex
plus a few keys required to support BibJSON, such as “namespaces”;
anything beyond the scope of BibTex should be added by using a
namespace.

**IUCr raw bibliography:**

```
<link rel="schema.DC" href="http://purl.org/dc/elements/1.1/" />
<link rel="schema.DCTERMS" href="http://purl.org/dc/terms/" />
<link rel="schema.prism" href="http://prismstandard.org/namespaces/1.2/basic/" />
<meta name="DC.source" content="urn:issn:1600-5368" />
<meta name="DC.rights" content="http://creativecommons.org/licenses/by/2.0/uk" />
<meta name="DC.creator" content="Zheng, L." />
<meta name="DC.creator" content="Hu, F." />
<meta name="DC.creator" content="Zeng, X.C." />
<meta name="DC.creator" content="Li, K.P." />
<meta name="DC.date" content="2011-04-01" />
<meta name="DC.identifier" content="doi:10.1107/S1600536811007148" />
<meta name="DC.publisher" content="International Union of Crystallography" />
<meta name="DC.link" content="http://scripts.iucr.org/cgi-bin/paper?cv5056" />
<meta name="DC.language" content="en" />
<meta name="DC.description" content="The title compound, C11H14N2O5, was synthesized by condensation of (RS)-2-aminosuccinic acid dimethyl ester with 2-trichloroacetylpyrrole at room temperature. The amide group is twisted by 7.4 (1)degrees from the plane of the pyrrole ring. In the crystal, molecules are linked by intermolecular N-H...O hydrogen bonds into chains extending along the c axis." />
<meta name="DC.type" content="text" />
<meta name="DC.title" content="rac-Dimethyl 2-(1H-pyrrole-2-carboxamido)butanedioate" />
<meta name="DCTERMS.abstract" content="The title compound, C11H14N2O5, was synthesized by condensation of (RS)-2-aminosuccinic acid dimethyl ester with 2-trichloroacetylpyrrole at room temperature. The amide group is twisted by 7.4 (1)degrees from the plane of the pyrrole ring. In the crystal, molecules are linked by intermolecular N-H...O hydrogen bonds into chains extending along the c axis." />
<meta name="prism.number" content="4" />
<meta name="prism.volume" content="67" />
<meta name="prism.publicationDate" content="2011-04-01" />
<meta name="prism.publicationName" content="Acta Crystallographica Section E: Structure Reports Online" />
<meta name="prism.issn" content="1600-5368" />
<meta name="prism.section" content="organic compounds" />
<meta name="prism.startingPage" content="752" />
<meta name="prism.rightsAgent" content="med@iucr.org" />
<meta name="prism.endingPage" content="752" />
<meta name="prism.eissn" content="1600-5368" />
<meta name="keywords" lang="en" content="" />
<meta name="ROBOTS" content="NOARCHIVE,NOINDEX" />
```

**IUCr bibJSON:**

```
[
    {
           "type" : "metadata",
           "namespaces" : {
                               "dc" : "http://purl.org/dc/elements/1.1/",
                               "prism" : "http://prismstandard.org/namespaces/1.2/basic/",
                               "bibo" : “http://purl.org/ontology/bibo/"
                           }
    },  
    {
           "url" : "http://scripts.iucr.org/cgi-bin/paper?cv5056",
           "author" : [
                           "Zheng, L.",
                           "Hu, F.",
                           "Zeng, X.C.",
                           "Li, K.P."
                      ],
           "abstract" : "The title compound, C11H14N2O5....",
           "journal" : "Acta Crystallographica Section E: Structure Reports Online"
           "bibo:issn" : "1600-5368",
           "bibo:doi" : "10.1107/S1600536811007148",
           "dc:rights" : "",
           "dc:date" : "2011-04-01",
           "dc:publisher" : "International Union of Crystallography",
           "dc:language" : "en",
           "dc:description" : "The title compound, C11H14N2O5...",
           "dc:title" : "rac-Dimethyl 2-(1H-pyrrole-2-carboxamido)butanedioate",
           "prism:number" : "4",
           "prism:volume" : "67",
           "prism:section" : "organic compounds"
           "prism:startingPage" : "752",
           "prism:endingPage" : "752",
           "prism:publicationDate" : "2011-04-01",
           "prism:eissn" : "1600-5368",
           "prism:rightsAgent" : "med@iucr.org",
    }
]
```

**Atmospheric Chemistry and Physics BibTex:**

```
@Article{acp-11-4679-2011,
AUTHOR = {Murphy, D. M. and Chow, J. C. and Leibensperger, E. M. and Malm, W. C. and Pitchford, M. and Schichtel, B. A. and Watson, J. G. and White, W. H.},
TITLE = {Decreases in elemental carbon and fine particle mass in the United States},
JOURNAL = {Atmospheric Chemistry and Physics},
VOLUME = {11},
YEAR = {2011},
NUMBER = {10},
PAGES = {4679--4686},
URL = {http://www.atmos-chem-phys.net/11/4679/2011/},
DOI = {10.5194/acp-11-4679-2011}
}
```

**Atmospheric Chemistry and Physics BibJSON:**

```
[
    {
           "type" : "article",
           "author" : [
                           "Murphy, D. M.",
                           "Chow, J. C",
                           "Leibensperger, E. M.",
                           "Malm, W. C.",
                           "Pitchford, M.",
                           "Schichtel, B. A.",
                           "Watson, J. G.",
                           "White, W. H."
                      ],
           "title" : "Decreases in elemental carbon and fine particle mass in the United States",
           "journal"  : "Atmospheric Chemistry and Physics",
           "volume" : "11",
           "year": "2011",
           "number" : "10",
           "pages" : "4679--4686",
           "url" : "http://www.atmos-chem-phys.net/11/4679/2011/",
           "doi" : "10.5194/acp-11-4679-2011",
    }
]
```

**J.ChemInf bibJSON (based on JChemInf RDF):**

```
[
    {
           "type" :"metadata",
           "namespaces" : {
                               "dc" : "http://purl.org/dc/elements/1.1/",
                               "dcterms" : "http://purl.org/dc/terms/",
                               "prism" : "http://prismstandard.org/namespaces/1.2/basic/"
                          },
    },
    {
           "url" : "http://www.jcheminf.com/content/3/1/17",
           "bibjson:fulltext" : "http://www.jcheminf.com/content/pdf/1758-2946-3-17.pdf",
           "abstract" : "http://www.jcheminf.com/content/3/1/17/abstract/",
           "title" : "ChemicalTagger: A tool for semantic text-mining in chemistry",
           "author" : [
                           "Lezan Hawizy",
                           "David Jessop",
                           "Nico Adams",
                           "Peter Murray-Rust"
                      ],
           "journal" : "Journal of Cheminformatics 2011 3:17",
           "dc:date" : "2011-5-16",  
           "dc:identifier" : "",
           "dc:publisher" : "Chemistry Central Ltd",
           "dc:rights" : "",
           "dc:language" : "en",
           "dc:format" : "text/html"
           "prism:publicationName" : "Journal of Cheminformatics"
           "prism:issn" : "1758-2946",
           "prism:publicationDate" : "2011-5-16",
           "prism:volume" : "3",
           "prism:number" : "1",
           "prism:startingPage" : "17",
           "prism:copyright" : "2011 Hawizy et al;",
           "prism:rightsAgent" : "reprints@biomedcentral.com",
    }
]
```

### 8.2 Medline sample record

```
## namespace prefixes used:
@prefix rdf:  .
@prefix rdfs:  .
@prefix owl:  .
@prefix dc:  .
@prefix dcat:  .
@prefix void:  .
@prefix bibo:  .
@prefix cito:  .
@prefix foaf:  .
@prefix skos:  .
@prefix opmv:  .
@prefix time:  .
@prefix xsd:  .

## metadata about the medline dataset the current record is in:
 a void:Dataset, dcat:Dataset ;
   ## licens terms of this (RDF) dataset
   dc:license  ;

   ## the RDF generation finished at this time
   dc:modified "2011-05-08T15:23:45Z"^^xsd:dateTime ;

   ## it came from this medline XML file
   dc:source "medline11n0421" ;

   ## which can be obtained here (in theory, not yet)
   void:dataDump  ;

   ## another way of expressing where it can be obtained
   dcat:distribution [
       dc:description "bzip2 compressed N-Quads" ;
       a dcat:Distribution ;
       dcat:accessURL 
   ] .

## information about the medline record in question:
 a foaf:Document;

   ## link back to the dataset
   dc:isPartOf  ;
   ## license terms of this metadata
   dc:license  ;

   ## various timestamps relating to
   dc:created "2000-06-28"^^xsd:dateTime ;
   dc:issued "2000-06-28"^^xsd:dateTime ;
   dc:modified "2004-11-17"^^xsd:dateTime ;

   ## provenance information
   dc:source "MEDLINE" ;
   opmv:wasGeneratedBy [
       a opmv:Process

       ## we used version 1.3 of the medline software, and the
       ## indicated source dataset
       opmv:used , [
           rdfs:label "medline11n0421"
       ] ;

       ## it was me that did this conversion
       opmv:wasControlledBy  ;

       ## at this time
       opmv:wasPerformedAt [
           a time:Instant ;
           time:inXSDDateTime "2011-05-08T15:23:45Z"^^xsd:dateTime
       ] ;
   ] ;

   ## this metadata record is about this article
   foaf:primaryTopic  .

## some further information about me, the person who did the conversion
 a foaf:Agent ;
   foaf:mbox  ;
   foaf:name "William Waites" .

## some further information about the software that was used

   dc:version "1.3" ;
   rdfs:label "Go Medline 1.3" .

## this is the journal that the article was published in
 a bibo:Journal ;
   dc:identifier "0077-8923", "7506858" ;
   dc:title "Annals of the New York Academy of Sciences" ;
   bibo:issn "0077-8923" ;

   ## we can use the ISO abbreviation to build up a concept
   ## of standard names for journals
   skos:prefLabel "Ann. N. Y. Acad. Sci." .

## information about the actual article
 a bibo:AcademicArticle ;
   dc:title "Paradoxical phosphorylation of skeletal muscle glycogen
             synthase by in vivo insulin in very lean young adult rhesus
             monkeys." ;
   dc:published "1999-18" ;
   dc:language "eng" ;

   ## this article was published in this issue...
   dc:isPartOf [
       a bibo:Issue ;
       bibo:volume "892" ;
       dc:published "1999-18" ;
       dc:spatial "UNITED STATES" ;
       ## .. which is part of this journal
       dc:isPartOf  ;
   ] ;
   ## .. on these pages
   dc:extent "247-60" ;

   ## and here are the authors
   dc:creator [
       a foaf:Person ;
       foaf:familyName "Ortmeyer" ;
       foaf:givenName "H K" ;
       foaf:name "Ortmeyer, H K"
   ], [
       a foaf:Person ;
       foaf:familyName "Bodkin" ;
       foaf:givenName "N L" ;
       foaf:name "Bodkin, N L"
   ], [
       a foaf:Person ;
       foaf:familyName "Hansen" ;
       foaf:givenName "B C" ;
       foaf:name "Hansen, B C"
   ] .
```

## REFERENCES

[1] *The Open Knowledge Foundation*. : http://okfn.org

[2] *JISC Open Bibliography project. Funded by Joint Information Systems Committee http://jisc.ac.uk. Thanks to the JISC Open Bibliography team: Peter Murray-Rust (University of Cambridge), Rufus Pollock (Open Knowledge Foundation, University of Cambridge), Ben O'Steen (Cottage Labs), David Flanders (JISC Program Manager)*. : http://openbiblio.net

[3] *Bibliographic Knowledge Network is supported by funding from the National Science Foundation (Award #0835851)*. : http://www.bibkn.org

[4] 2000. *MARC21 specifications for record structure, character sets, and exchange media*. Library of Congress Network Development and MARC Standards Office. : http://www.loc.gov/marc/specifications/

[5] Ellen Gredley,Alan Hopkinson. 1990. *Exchanging bibliographic data: MARC and other international formats*. Library Association Publishing, London. ISBN 888022581. : http://eprints.mdx.ac.uk/2930/

[6] *BibTex bibliographic format*. : http://www.bibtex.org

[7] *RIS file format*. : http://www.refman.com/support/risformat\_intro.asp

[8] *XML specification*. : http://www.w3.org/TR/xml/

[9] *JavaScript Object Notation format*. : http://www.json.org/

[10] Fielding, Roy Thomas. 2000. *Architectural Styles and the Design of Network-based Software Architectures*. Doctoral dissertation. University of California Irvine. : http://www.ics.uci.edu/~fielding/pubs/dissertation/top.htm

[11] *NoSQL database defintion*. : http://nosql-database.org/

[12] *Apache CouchDB project*. : http://couchdb.apache.org/

[13] *Apache Lucene project*. : http://lucene.apache.org/

[14] *Apache SOLR project*. : http://lucene.apache.org/solr/

[15] *Elastic Search project*. : http://www.elasticsearch.org/

[16] *The CERN Library publishes its book catalogue as Open Data*. : http://library.web.cern.ch/library/Library/announcement.html

[17] *Library of Congress Subject Headings, published as Linked Open Data*. : http://id.loc.gov/authorities/subjects.html

[18] *UK government provides open data*. : http://data.gov.uk/

[19] *Libraries in Cologne open up bibliographic data*. : http://blog.okfn.org/2010/03/15/libraries-in-cologne-open-up-bibliographic-data/

[20] Garfield, Eugene. 1986. *Essays of an information scientist*. ISI Press, Philadelphia, PA.

[21] E. Garfield. *Using the impact factor*. Current Contents, July 18 1994. : http://researchanalytics.thomsonreuters.com/essays/usingimpactfactor/

[22] Richard K Belew. 2005. *Scientific impact quantity and quality: Analysis of two sources of bibliographic data*. : http://arxiv.org/abs/cs/0504036

[23] *The open definition*. : http://opendefinition.org

[24] *The LaTeX project*. : http://www.latex-project.org/

[25] *The Dublin Core Metadata Initiative*. : http://dublincore.org/

[26] *Knowledge 4 All project*. : http://www.k4all.org/

[27] *The WikiPedia project*. : http://www.wikipedia.org/

[28] *arXiv.org e-print archive*. : http://arxiv.org/

[29] *Research papers in Economics*. : http://repec.org/

[30] *BibSonomy social bookmark and publication system*. : http://www.bibsonomy.org/

[31] *PhilPapers: online research in philosophy*. : http://philpapers.org/

[32] *The DBPL Computer Science Bibliography*. : http://dblp.uni-trier.de/

[33] *CiteULike service for managing and discovering scholarly references*. : http://www.citeulike.org/

[34] *Connotea: free online reference management for all researchers, clinicians and scientists*. : http://www.connotea.org/

[35] *Zotero tool to collect, organize, cite and share research sources*. : http://www.zotero.org/

[36] *Mendeley academic reference management software for researchers*. : http://www.mendeley.com/

[37] *International Union of Crystallography*. : http://www.iucr.org/

[38] *BioMed Central: The Open Access publisher*. : http://www.biomedcentral.com/

[39] *PubMed: U.S. National Library of Medicine. National Institute of Health*. : http://www.ncbi.nlm.nih.gov/pubmed/

[40] *AuthorClaim registration service*. : http://authorclaim.org/

[41] *The Freelib project*. : http://3lib.org/

[42] *SciPlore exploring science service*. : http://www.sciplore.org/

[43] K.G. Saur. 1998. *Functional Requirements for Bibliographic Records, Final Report / IFLA Study Group on the Functional Requirements for Bibliographic Records*. UBCIM Publications, New Series ; v. 19. : http://www.ifla.org/VII/s13/frbr/frbr.htm

[44] *The Bibliographic Ontology*. : http://bibliontology.com/

[45] Karen Coyle. *Understanding the Semantic Web: Bibliographic Data and Metadata*. : http://www.alatechsource.org/library-technology-reports/understanding-the-semantic-web-bibliographic-data-and-metadata

[46] D Beckett. 2004. *RDF/XML syntax specification (revised)*. : http://www.w3.org/TR/2004/REC-rdf-syntax-grammar-20040210

[47] Jim Pitman,Nitin Borwankar. 2009. *BibJSON Bibliographic Record Specification*. : http://www.bibkn.org/bibjson/index.html

[48] *The OpenStreetMap project*. : http://www.openstreetmap.org/

[49] *ChemSpider: The free chemical database*. : http://www.chemspider.com/

[50] *The BibSOUP project*. : http://bibserver.okfn.org/bibsoup/

[51] L. Page,S. Brin,R. Motwani,T. Winograd. 1999. *The PageRank Citation Ranking: Bringing Order to the Web*. : http://ilpubs.stanford.edu:8090/422/

[52] Richard Cyganiak. 2007. *The Linked Open Data Cloud*. : http://richard.cyganiak.de/2007/10/lod/

[53] *Endnote reference management software*. : http://www.endnote.com

[54] *The CrossRef DOI resolver*. : http://www.crossref.org/

[55] G. Bilder. 2011. *Content negotiation for crossref DOIs*. : http://www.crossref.org/CrossTech/2011/04/content\_negotiation\_for\_crossr.html

[56] Peter Murray-Rust. *Bibliographic data is open*. : http://blogs.ch.cam.ac.uk/pmr/2011/02/04/bibliographic-data-is-open/

[57] Peter Murray-Rust,Karen Coyle,Mark MacGillivray,Ben O'Steen,Jim Pitman,Adrian Pohl,Rufus Pollock,William Waites. *The Open Bibliographic principles*. : http://openbiblio.net/principles

[58] *CiteSeer Scientific Literature Digital Library and Search engine*. : http://citeseerx.ist.psu.edu/

[59] *U.S. National Library of Medicine MEDLINE factsheet*. : http://www.nlm.nih.gov/pubs/factsheets/medline.html

[60] *SKOS - Simple Knowledge Organization System*. : http://www.w3.org/2004/02/skos/

[61] T. Berners-Lee,R. Fielding,L. Masinter. 1998. *Uniform resource identifiers (URI): Generic syntax. IETF RFC 2396*. : http://www.ietf.org/rfc/rfc2396

[62] *The British Library*. : http://bl.uk

[63] 2010. *British Library to share millions of catalogue records*. : http://pressandpolicy.bl.uk/Press-Releases/British-Library-to-share-millions-of-catalogue-records-43b.aspx

[64] *U.C. Berkeley Mathematical faculty list*. : http://math.berkeley.edu/people\_faculty.html

[65] *Bibliographic Knowledge Network: People*. : http://people.bibkn.org

[66] *Bibliographica*. : http://bibliographica.org

[67] *The OpenBiblio software repository*. : https://bitbucket.org/okfn/openbiblio

[68] Mark MacGillivray. *Getting open bibliographic data from PMC*. : http://openbiblio.net/2011/05/03/getting-open-bibliographic-data-from-pmc/

[69] William Waites. 2011. *Medline RDF*. : http://eris.okfn.org/ww/2011/05/medline/

[70] *Medline dataset available on CKAN*. : http://ckan.net/package/medline

[71] AJ Wakefield,SH Murch,A Anthony,J Linnell,DM Casson,M Malik,M Berelowitz,AP Dhillon,MA Thomson,P Harvey,A Valentine,SE Davies,JA Walker-Smith. 1998. *RETRACTED: Ileal-lymphoid-nodular hyperplasia, non-specific colitis, and pervasive developmental disorder in children.* DOI 10.1016/S0140-6736(97)11096-0. : http://dx.doi.org/10.1016/S0140-6736(97)11096-0

[72] David Shotton. 2010. *CiTO, the citation typing ontology*. DOI 10.1186/2041-1480-1-S1-S6. : http://dx.doi.org/10.1186/2041-1480-1-S1-S6

[73] Tatiana De La O. 2011. *Bibliographica gadget in Wikipedia*. : http://openbiblio.net/2011/06/06/bibliographica-gadget-in-wikipedia/

[74] Tatiana De La O. 2011. *Collections in Bibliographica*. : http://openbiblio.net/2011/06/12/collections-in-bibliographica/

[75] Mark MacGillivray. 2011. *Bibliographica and Edinburgh International Science Festival*. : http://openbiblio.net/2011/04/11/bibliographica-and-edinburgh-international-science-festival/

[76] *The GeoJSON specification*. : http://geojson.org/geojson-spec.html

[77] *Scholarly HTML*. : http://scholarlyhtml.org/

[78] *Schema.org*. : http://schema.org/

[79] *The BibServer project*. : http://bibserver.okfn.org/bibserver
